# Hepatitis E in southern Vietnam: Seroepidemiology in humans and molecular epidemiology in pigs

**DOI:** 10.1111/zph.12364

**Published:** 2017-06-09

**Authors:** A. Berto, H. A. Pham, T. T. N. Thao, N. H. T. Vy, S. L. Caddy, R. Hiraide, N. T. Tue, I. Goodfellow, J. J. Carrique-Mas, G. E. Thwaites, S. Baker, M. F. Boni

**Affiliations:** 1 Wellcome Trust Major Overseas Programme, Oxford University Clinical Research Unit, Ho Chi Minh City, Vietnam; 2 Nuffield Department of Clinical Medicine, Centre for Tropical Medicine and Global Health, Oxford, UK; 3 UK Division of Virology, Department of Pathology, University of Cambridge, Cambridge, UK; 4 The Department of Medicine, University of Cambridge, Cambridge, UK; 5 Center for Infectious Disease Dynamics, Department of Biology, Pennsylvania State University, State College, PA, USA

**Keywords:** hepatitis E virus, human, pigs, prevalence, seroprevalence, Vietnam, zoonosis

## Abstract

Viral pathogens account for a significant proportion of the burden of emerging infectious diseases in humans. The Wellcome Trust-Vietnamese Initiative on Zoonotic Infections (WT-VIZIONS) is aiming to understand the circulation of viral zoonotic pathogens in animals that pose a potential risk to human health. Evidence suggests that human exposure and infections with hepatitis E virus (HEV) genotypes (GT) 3 and 4 results from zoonotic transmission. Hypothesising that HEV GT3 and GT4 are circulating in the Vietnamese pig population and can be transmitted to humans, we aimed to estimate the seroprevalence of HEV exposure in a population of farmers and the general population. We additionally performed sequence analysis of HEV in pig populations in the same region to address knowledge gaps regarding HEV circulation and to evaluate if pigs were a potential source of HEV exposure. We found a high prevalence of HEV GT3 viral RNA in pigs (19.1% in faecal samples and 8.2% in rectal swabs) and a high HEV seroprevalence in pig farmers (16.0%) and a hospital-attending population (31.7%) in southern Vietnam. The hospital population was recruited as a general-population proxy even though this particular population subgroup may introduce bias. The detection of HEV RNA in pigs indicates that HEV may be a zoonotic disease risk in this location, although a larger sample size is required to infer an association between HEV positivity in pigs and seroprevalence in humans.

## 1 Introduction

Emerging infectious diseases have an important impact on human health. It is well established that viruses account for a significant proportion of emerging infections in humans and the majority are have a zoonotic origin, as highlighted by the recent Ebola epidemic in Africa and the repeated MERS CoV outbreaks in the Middle East. Hepatitis E virus (HEV) is a public health concern, as it causes an estimated 20 million human infections annually, with over three million symptomatic HEV cases and 56,600 deaths worldwide (Kamar et al., 2012). Mortality in immunocompromised patients and pregnant women can approach 25% for those infected with genotype 1 (Kamar et al., 2012). HEV infections are responsible for >50% of cases of acute viral hepatitis in endemic countries (Kamar et al., 2012). HEV is generally associated with a self-limiting hepatitis, as the infection clears within 3 months from onset of symptoms. Recently, it was observed that infection with HEV GT3 can become chronic in immunocompromised patients, such as organ transplant recipients or those infected with HIV (Galante et al., 2015).

HEV is the sole member of the *Hepeviredae* family of the *Orthohepevirus A* genus. It has only one serotype and four genotypes (GTs), although a reclassification into seven GTs has been proposed (Smith et al., 2016). Of the four genotypes, GT1 and GT2 are able to sustain human-to-human transmission and sporadically cause large human outbreaks in endemic regions due to faecal contamination of the water supply. Genotypes 3 and 4 are typically zoonotic; infection can occur from eating undercooked pork or venison, via direct exposure to animal faeces, and sporadically through blood transfusion or liver transplantation (Berto et al., 2013; Christou & Kosmidou, 2013; Lu, Li, & Hagedorn, 2006; Renou et al., 2007; Shen et al., 2007; Sridhar, Lau, & Woo, 2015; Tedder et al., 2017). There is now evidence that HEV GTs 3 and 4 infections mainly originate in swine, and local zoonotic transmission of HEV has been recorded worldwide (Christou & Kosmidou, 2013).

Recent initiatives to increase and improve the surveillance for HEV disease in humans have shown that it may be more common than hepatitis A; however, the true incidence of HEV infections in humans is still not known (Kamar et al., 2012; da Silva et al., 2012). It has been shown that seroprevalence of HEV in human populations varies between 10% and 50% in blood donors, depending on the geographical location (Dalton et al., 2008; Scotto et al., 2012; Slot et al., 2013; Vollmer et al., 2012). Furthermore, it is possible that the majority of patients with unexplained and undiagnosed hepatitis may be HEV positive as HEV is generally not considered as a potential cause especially in developing countries, due to the lack of diagnostic tests (Kamar et al., 2012; da Silva et al., 2012). The seroprevalence of HEV in pigs in various European countries varies between 12.5% and 80% (Berto et al., 2012; Mccreary et al., 2008), and antibodies against HEV have also been detected in several other species worldwide, such as rabbit, rats, cattle, sheep, goats, chickens and dogs (Drexler et al., 2012; Johne et al., 2010; Sun et al., 2004).

To date, there are only two published studies describing the circulation of HEV in Vietnam. One reported a HEV seroprevalence rate of 2% in 90 patients with elevated alanine aminotransferase (ALT) (Buchy et al., 2004), while the second reported that nine of 141 human serum samples obtained from patients with acute sporadic hepatitis in Hanoi were infected with HEV genotype 4 (Dieu Ngan et al., 2013).

Vietnam, along with other Southeast Asian countries, is considered a global hotspot for zoonotic infections (Jones et al., 2008). The WT-VIZIONS (Wellcome Trust-Vietnamese Initiative on Zoonotic Infections) programme is aiming to gather data on the circulation of viral zoonotic pathogens in animals that pose a risk to human health. Hypothesising that HEV GT3 and GT4 are common zoonotic pathogens in Vietnam, we aimed to estimate (i) the HEV seroprevalence in a hospital-attending population, as a proxy for the general population, (ii) the HEV seroprevalence in individuals working in close contact with pigs (farmers, family members of farmers, animal workers, veterinarians and abattoir workers) and (iii) the prevalence of HEV infection in pigs.

All collected farm samples (faeces/rectal swabs and human plasma) were collected from Dong Thap province (southern Vietnam). Furthermore, we retrospectively tested for presence of anti-HEV IgG in a hospital population sample of 1,726 human serum samples collected between 2009 and 2014 from Dong Thap.

## 2 Material and Methods

### 2.1 Study design

The WT-VIZIONS is a descriptive observational farm-based and community-based study of zoonotic infection, diseases in domestic livestock and pathogens circulating among individuals with significant occupational or residential exposures to domestic livestock, wildlife and/or animal products. The study enrolled 300 cohort members from approximately 60 different sites in Dong Thap province. The WT-VIZIONS study design aimed at having an average of 3–5 cohort members per farm and 10–15 cohort members per abattoir/market (Carrique-Mas et al., 2015; Rabaa et al., 2015).

Selection of Dong Thap province for this substudy was coordinated with a complementary long-term seroepidemiology study from Dong Thap Provincial Hospital (DTPH), with residual samples collected from the hospital haematology laboratory; this was part of an on-going seroepidemiology study being conducted in southern Vietnam (Boni et al., 2013; Nhat et al., 2016). Additionally, Dong Thap province was selected because the high density of domestic livestock and/or farmed wildlife populations, the diversity of local agro-ecosystems, and pre-existing frameworks for collaborative research between Oxford University Clinical Research Unit and Sub-Departments of Preventive Medicine and Animal Health (PMC and SDAH, respectively).

The VIZIONS study team, working with the SDAH, identified the individual farms and abattoirs within Dong Thap province. The Provincial Coordinator (PC) of Dong Thap organised a first site visits to confirm whether inclusion criteria were met. At each study site, the person with primary management responsibility was identified (farmer, farm manager or abattoir manager), informed of the study objectives, and asked to obtain written informed consent to sample animals and human blood. The study sites were included in the study if they fulfilled the following criteria: permission from local authorities (Peoples’ Committee and institutional management bodies), farmers provided written permission, minimum two consenting participants per site and access to a cell phone with texting capacity. Sites were excluded if they were difficult to reach, if the participant was less than 1 year of age, or if the participant had any known immunosuppressive condition or ongoing receipt of any immunosuppressive therapy.

The study was approved by the University of Oxford Tropical Research Ethics Committee (OxTREC No. 0109) and the Scientific and Ethical Committee of the Hospital for Tropical Diseases in Ho Chi Minh City. Written informed consent was required from patients and parents or legal guardians if children under age of 16 prior to participation in the study.

### 2.2 Sample collection

Pig faeces samples from 774 pigs were collected on 104 farms during 2012, while 293 pig rectal swabs were collected in 2013–2014 from 29 farms. From each farm up to 15 individual animals were sampled. As this study was a part of the WT-VIZIONS project, which aimed to assess the circulation of many different pathogens in addition to HEV, the number of animals to sample from each species was a function of the relative number of animals of each species present on the farm. For the above reason, a scoring system was developed to assign different weight to each species and calculate the number of individuals to sample. Therefore, for each farm between 1 and 15, pigs were randomly sampled depending on the total number of different animals species present on the farm (Carrique-Mas et al., 2015; Rabaa et al., 2015). The total number of pigs for each farm was not recorded. The age of the pigs varied between 6 weeks and 4 years, but the precise age of the individual sampled pig was not recorded, although the majority of the animals were adult pigs. In all farms, samples of a minimum of 1 g of faeces were collected aseptically in a sterile plastic container and resuspended in 1 ml of viral transport medium (VTM); rectal swabs were placed in tubes containing 1 ml of VTM. The collected samples were maintained at 4°C (max. 24 hr), subsequently transported to OUCRU and frozen at −20°C until processing.

Human plasma samples were collected from 60 sampling sites: the 29 farms sampled in 2013–2014 from which pig rectal swabs were tested, 23 additional farms from which no pig rectal swabs were available, three rodent trading sites, two poultry markets, two pheasant trading sites and one abattoir. An average of five human plasma samples were collected from each site (281 in total), and the plasma sampling on the 29 farms with pig rectal swabs was concurrent with the pig sampling. At enrolment, participants were asked to consent to a 10 ml whole blood specimen collection (5 ml for children aged <5 years). Blood was allowed to clot at room temperature, transported at ambient temperature and centrifuged within 24 hr. The primary coordinator coordinated the storage and shipment of human specimen materials to OUCRU laboratory in HCMC. All specimens were separated into three equal aliquots and preserved at −80°C until processing.

Serum samples from the hospital population were collected from DTPH from the hospital haematology laboratory, as part of an on-going seroepidemiology study currently being conducted in southern Vietnam. In this study, 200 samples are collected at each collection time point (between three and six per year), in order to allow for the lower range of seroprevalences to be inferred accurately; with this design, a 5% seroprevalence can be inferred with a 95% confidence interval from 2.4% to 9.0%. In each collection, a minimum of 50 samples were required from each of the age bands 0–19, 20–39 and 40+. For the subsample used in this study, 1,726 samples were selected. The sampling design was approximately 300 per year from 2009 to 2014, with a 1:1 gender ratio. Thirty samples were chosen from each of the age bands 10–20, 20–30, 30–40 and 40–50. Fifteen samples were chosen from the age bands 0.5–10 and 50–80. The entire sample set is smaller than the intended 1,800 as some age groups did not have enough samples. Each collected specimen contained approximately 0.5 ml of serum. Samples for this seroepidemiology study are stored at −20°C and transported on dry ice twice yearly to Ho Chi Minh City where they are stored at −20°C as part of a central serum bank for southern Vietnam (Boni et al., 2013; Nhat et al., 2016; Thuy et al., 2017).

### 2.3 Seroepidemiology and molecular epidemiology methods

**Figure 1:**
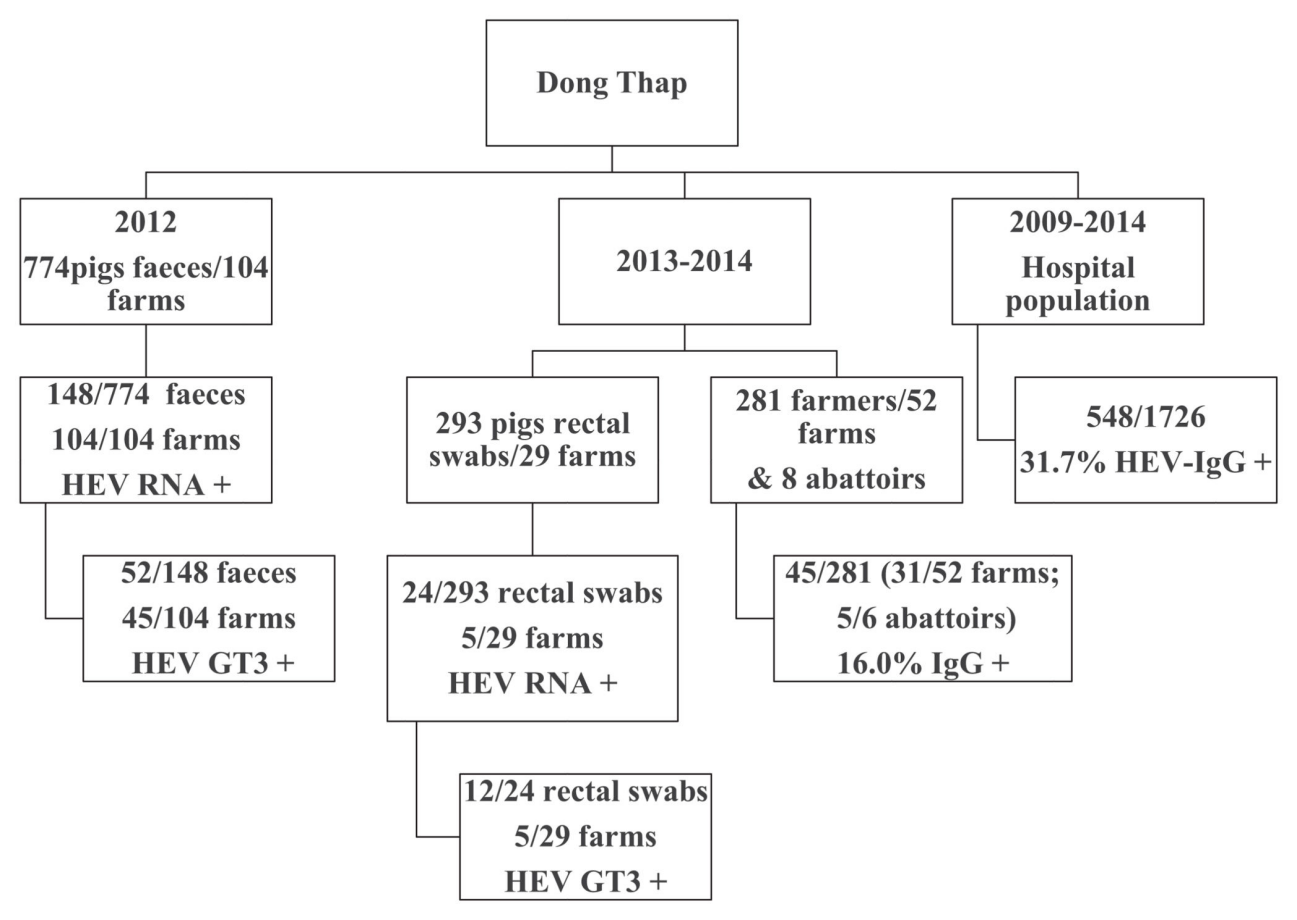
Study design diagram and results summary

Plasma from the farmers and farmers’ family members, veterinarians, animal workers and abattoir workers (*n* = 281, 52 farms plus eight other sites) and serum from the hospital population (*n* = 1,726) from Dong Thap (Figure 1) were screened for anti-HEV antibody (IgG) using the Wantai ELISA kit (Sanbio, China), following the manufacturer’s instructions (Izopet et al., 2015).

All faecal samples and rectal swabs from pigs were screened for HEV RNA using quantified PCR (qPCR) to detect HEV RNA as described by Jothikumar, Cromeans, Robertson, Meng, and Hill (2006). The qPCR-positive samples were amplified using a nested PCR targeting a 300 bp fragment of ORF2 as previously described by Li et al. (2009). All PCR amplicons (300 bp) were visualised on 2% agarose gels under ultraviolet (UV) light and sequenced by Sanger sequencing using an ABI 3700 sequencer in both forward and reverse directions using the primers from the second round nested PCR. The HEV sequences were aligned with a subset of publicly available reference sequences from all seven recently new proposed classification of HEV genotypes (Smith et al., 2016), yielding a 37-reference sequences data set (both ORF2 and whole genome) and 64 Vietnamese sequence data set from pigs (300 bp). All sequences were aligned with MEGA6. Maximum-likelihood phylogeny was inferred using RAxML v8.2.8 (Stamatakis, 2014) with 100 bootstrap replicates. The tree was visualised and annotated using FigTree (version 1.4.1).

### 2.4 Statistical analysis

A standard LOESS curve (local polynomial regression fitting; smoothing parameter = 0.75) was used to construct an age-seroprevalence curve with 95% confidence intervals. A Spearman rank correlation coefficient was used to determine the correlation between the HEV RNA-positive pigs and the HEV seropositivity of farmers. All confidence intervals in the text are presented as exact binomial confidence intervals.

## 3 Results

The pig faecal samples (*n* = 774; 104 farms; 2012) and rectal swabs (*n* = 293; 29 farms; 2013–2014) from Dong Thap were screened by qPCR. The number of samples tested for each farm varied from 1 to 15 depending on the size of each farm. HEV RNA was detected by qPCR in 148 of 774 faecal samples (19.1% prevalence; 95%CI: 16.4%-22.1%), with 45 of 104 farms having at least one positive animal. Twenty-four of 293 rectal swabs were HEV RNA positive by qPCR (8.2% prevalence, 95% CI: 5.3%-11.9%) with five of 29 farms having at least one positive animal (Table 1, Figure 1). The fraction of positive pigs on each of the five farms where samples were collected during 2013–2014 was 2 of 8, 11 of 13, 2 of 5, 6 of 7 and 3 of 5. The total number of pigs on each farm from samples collected during 2012 was not recorded, and the HEV prevalence for each individual farm could not be estimated.

**Figure 2:**
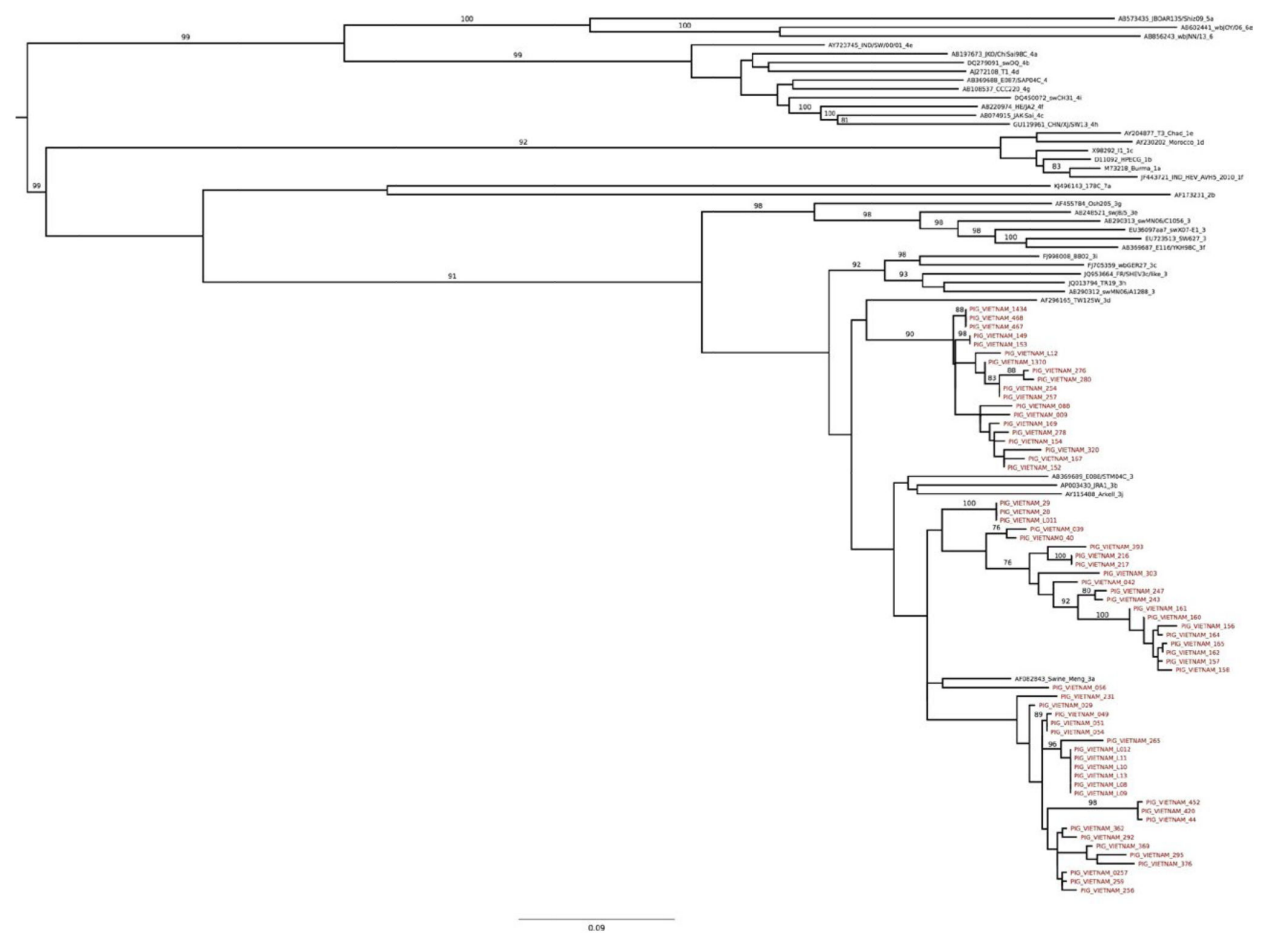
The phylogenetic relationships of hepatitis E virus sampled from Vietnamese pigs. Maximum-likelihood phylogeny constructed using RNA sequences from 64 Vietnamese pigs, and 37 reference sequences from the conserved region within the HEV ORF2 region (300 bp) from all four genotypes accessed from GenBank. Scale bar indicates the number of substitutions per site. Bootstrap support values are shown for nodes with ≥70% bootstrap support. The tree is mid-point rooted for clarity. Sequences from Vietnamese pigs are labelled in red. (Abbreviations: sw; swine, wb; wild boar; h; human). [Colour figure can be viewed at wileyonlinelibrary.com]

Of these HEV RNA-positive specimens, sequences suitable for phylogenetic analysis were obtained for 52 of 148 faecal samples collected in 2012 and in 12 of 24 rectal samples collected between 2013 and 2014 from Dong Thap (Table 1, Figure 1). All 64 sequences obtained from the HEV pig samples (GenBank accession numbers from KX092229 to KX092292) were found to belong to GT3 and clustered into three main clades (Figure 2). All Vietnamese pig sequences clustered separately from the previously identified GT3 sequences circulating worldwide and the subgenotypes did not cluster with any previously known subgenotype (Figure 2).

**Figure 3:**
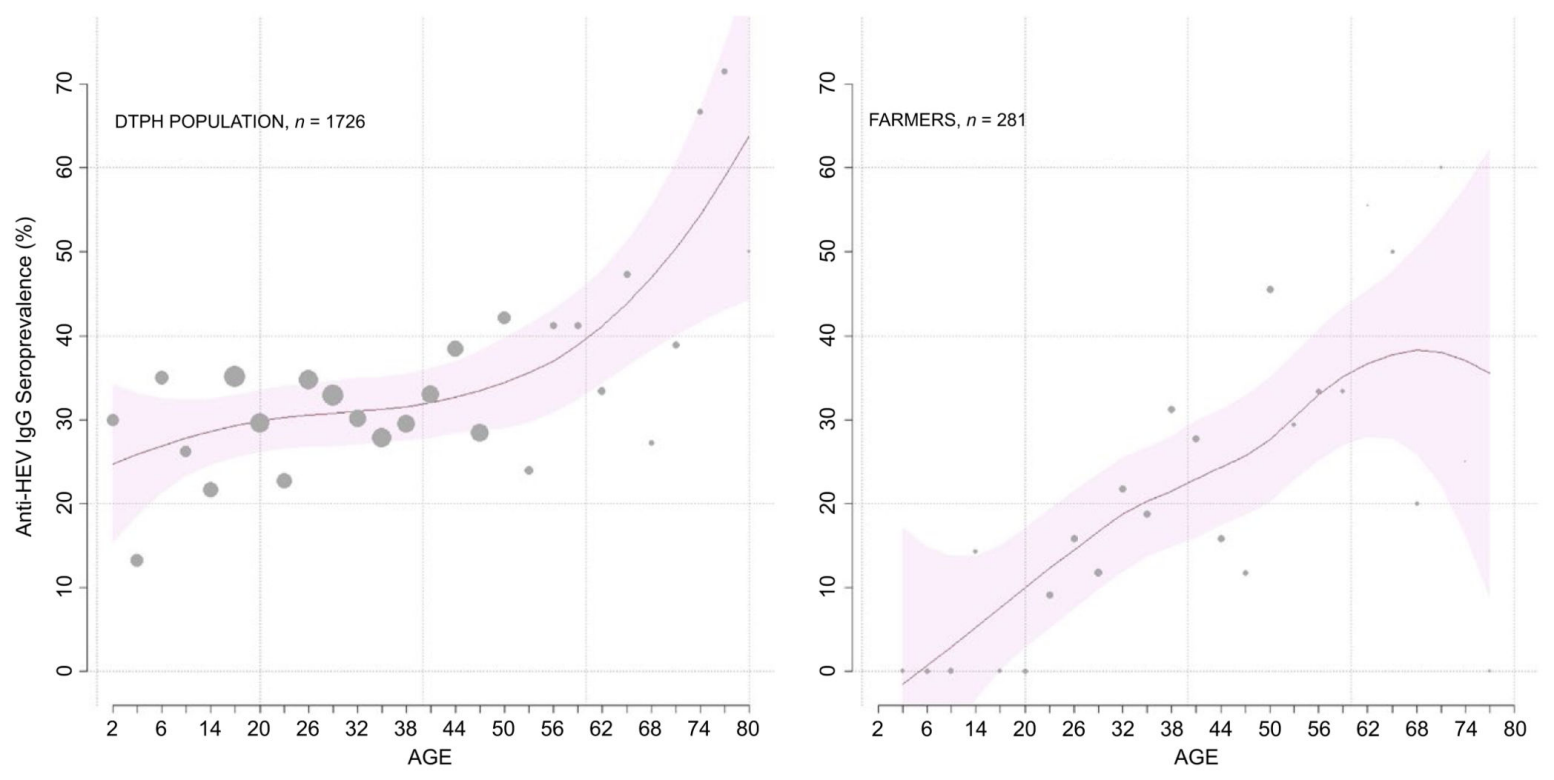
Seroprevalence in both general population and framers cohort groups analysed in this study. The graphs represent the total seroprevalence observed in the general population (left panel) and in the farmer cohort (right panel) stratified by age. *Y*-axis shows the seroprevalence percentage of people anti-HEV IgG positive, while the *X*-axis indicates the age groups; individuals were grouped into 3-year-age bands for maximum informativeness and clarity. The red line shows the inferred seroprevalence curve, and the pink shaded area shows the 95% confidence band. The grey dots show the seroprevalence for each 3-year-age group, and the size of the dot is proportional to the sample size. [Colour figure can be viewed at wileyonlinelibrary.com]

The anti-HEV IgG unadjusted seroprevalence was lower in the farmer cohort (16.0%; 95% CI: 11.7%–20.2%) than the general population (31.7%, 95% CI: 29.5%–33.8%) in Dong Thap (Table 1, Figure 1). There was no difference in seroprevalence by gender in either sample set. Although the seroprevalence in both populations increased with age, the prevalence of anti-HEV IgG was higher in children in the hospital population than in children enrolled in the farm cohort study (Figure 3). Breaking the comparisons into 10-year-age bands, the hospital population had higher HEV seroprevalence for individuals aged 0–20 (both *p* < .001; one-way test on ELISA optical density); HEV seroprevalence was indistinguishable between the two groups when considering individuals aged >20 years (all *p* > .06) (Figure 3). HEV seroprevalence did not vary through time in the hospital population cohort.

Due to the small sample size (*n* = 29) of farms with both molecular diagnostics on pigs and farmer plasma samples, there was insufficient statistical power to determine if there was a positive correlation between HEV RNA-positive pigs and seropositive farmers (Spearman *p*-value = .38).

## 4 Discussion

This study aimed to measure the prevalence of faecal HEV shedding on pig farms and the seroprevalence of HEV in the human population in southern Vietnam (Dong Thap province). We report an observed prevalence of HEV RNA in pigs in southern Vietnam that is similar to countries in Europe and Asia (Liu et al., 2015). The HEV prevalence observed in pig rectal swab samples was 8.2%, which is lower in comparison with previous studies, but the sensitivity of PCR screening is considerably lower using rectal swabs than faecal samples. We additionally found that all sequences obtained from pig samples were HEV GT3. Despite all samples being collected in the same province, these data show that HEV sequences clustered into three distinct clades within GT3, and the clusters were not associated with the farm or year of collection. Further, although the sequences clustered with HEV GT3, they did not cluster with previously known subgenotypes. These data confirm the substantial genetic variability that exists within the various HEV genotypes (Cao, Dianjun, & Meng, 2012).

The HEV seroprevalence identified in the farmer cohort (16.0%) was comparable to other Asian and European countries (Drexler et al., 2012; Johne et al., 2010; Sun et al., 2004). The retrospective serology from the general population identified an unadjusted seroprevalence of 31.7%. Despite using the same ELISA as other studies, this figure is lower when compared to previous data from Vietnam (42%) and other endemic regions such as Nepal (47.1%) and Bangladesh (49.8%) (Izopet et al., 2015; Tran et al., 2003); nevertheless, the results show that a large number of individuals in southern Vietnam are exposed to HEV.

While the participants studied in the farmer cohort were healthy individuals, the samples from the “general-population proxy” are best described as a sample from the potentially hospital-attending population. The same type of comparison is performed in other epidemiological studies where blood donors, for logistic reasons (e.g., easier recruitment), are recruited as a general-population proxy even though this particular population subgroup may introduce bias. As our study assessed the HEV seroprevalence in the hospital-attending population, it is important to highlight this distinction because some socioeconomic or occupational groups may have more reason to attend a hospital than others. Thus, these samples may represent individuals that have higher disease risk or vulnerability, to HEV or to infections in general, and hence may not be representative of the population at large. In particular, children in this sample are likely to be more vulnerable than children in a true general-population sample. The observed low seroprevalence in young children from the farmer cohort is in agreement with previous published data from endemic and non-endemic countries, such as Nepal, Bangladesh and France (Izopet et al., 2015), contrary to what was observed in the same age group in the hospital population sample in Dong Thap.

Despite not knowing the exact representativeness of the general-population samples, we believe that the results of this study provide unique and valuable insights into the epidemiology of HEV in the south of Vietnam that could be used to investigate associations of HEV seroprevalence and putative risk factors by a nationwide cross-sectional study. In particular, despite HEV GT3 being a zoonotic infection, a higher seroprevalence was not found in adult farmers than in other adults. This suggests that additional HEV risk factors need to be identified and evaluated. Confounders associated with the high HEV seroprevalence detected in both farmers and the hospital-attending population may be due to other risk factors rather than just direct contact with pigs. One potential risk factor might be consumption of internal pig organs, which is common in Vietnam. Other risk factors associated with high seroprevalence in human may be due to household flooding, drinking contaminated water or poor household hygiene. Future population-based studies, using random controls chosen by geographic area, should be performed to confirm or contradict these results. Farmers do not have a higher HEV seroprevalence than adults in the DTPH sample; however if they did, the relative risk of zoonotic HEV genotypes (i.e., GT3 or GT4) would be impossible to estimate using this seroprevalence assay.

Our initial hypothesis was that individuals living on pig farms would have higher HEV seroprevalence than the general population. We did not find evidence to support this hypothesis. However, our study had certain limitations; first no viral characterisation was performed in the two human cohorts, as no individuals presented with symptoms of liver diseases or jaundice during the study. Therefore, we could not link human HEV genotypes to pig HEV genotypes; only presence of anti-HEV IgG was measured, and direct evidence of HEV zoonotic infection was not identified. Furthermore, it was not possible to determine the genotype of HEV exposure in humans because HEV has only one serotype, and the ELISA cannot distinguish the different GTs. Our farm-based individuals work and live in close contact with pigs, and Dong Thap is a semi-rural region with a high density of pig farms; therefore, we speculate that the majority of the individual anti-HEV IgG-positive individuals have been exposed to HEV GT3 rather than other HEV genotypes (only HEV GT3 was identified in the pigs in our sample).

In conclusion, this study demonstrates for the first time that HEV is circulating in the pig population in southern Vietnam, and also that the human population in the same location has high seroprevalence to HEV. Therefore, this study provides data that may be used for HEV risk factor assessment to prevent and reduce HEV infection and transmission. More studies, incorporating testing blood donors or an alternative healthy general-population sample, are required to better understand HEV dynamics in human and pig populations in Vietnam in order to assess the implication for human health.

## Figures and Tables

**Table 1:** Summary of the total HEV prevalence and seroprevalence observed in pigs and human in the southern Vietnam

	Seroprevalence Pos/Tot (%)	Prevalence Pos/Tot (%)	95% CI
Pigs 2012		148/774 (19.1)	16.4%–22.1%
Pigs 2013–2014		24/293 (8.2)	5.3%–11.9%
Farmers	45/281 (16.0)		11.7%–20.2%
Hospital population	548/1726 (31.7)		29.5%–33.8%

## Impacts

- This study demonstrates that HEV is widely circulating in pig and human populations in southern Vietnam.
- The prevalence of HEV GT3 in swine farms in southern Vietnam was estimated at 19.1% and 8.2% in 2012 and in 2013–2014, respectively.
- The observed, unadjusted HEV seroprevalence in a population of farmers and a hospital population was 16.0% and 31.7%, respectively.
